# Measuring auditory selective attention using frequency tagging

**DOI:** 10.3389/fnint.2014.00006

**Published:** 2014-02-05

**Authors:** Hari M. Bharadwaj, Adrian K. C. Lee, Barbara G. Shinn-Cunningham

**Affiliations:** ^1^Center for Computational Neuroscience and Neural Technology, Boston UniversityBoston, MA, USA; ^2^Athinoula A. Martinos Center for Biomedical Imaging, Massachusetts General HospitalCharlestown, MA, USA; ^3^Department of Biomedical Engineering, Boston UniversityBoston, MA, USA; ^4^Department of Speech and Hearing Sciences, Institute for Learning and Brain Sciences, University of WashingtonSeattle, WA, USA

**Keywords:** auditory selective attention, auditory steady-state response, cortical gain control, frontal eye-fields, functional connectivity, frequency tagging, source localization

## Abstract

Frequency tagging of sensory inputs (presenting stimuli that fluctuate periodically at rates to which the cortex can phase lock) has been used to study attentional modulation of neural responses to inputs in different sensory modalities. For visual inputs, the visual steady-state response (VSSR) at the frequency modulating an attended object is enhanced, while the VSSR to a distracting object is suppressed. In contrast, the effect of attention on the auditory steady-state response (ASSR) is inconsistent across studies. However, most auditory studies analyzed results at the sensor level or used only a small number of equivalent current dipoles to fit cortical responses. In addition, most studies of auditory spatial attention used dichotic stimuli (independent signals at the ears) rather than more natural, binaural stimuli. Here, we asked whether these methodological choices help explain discrepant results. Listeners attended to one of two competing speech streams, one simulated from the left and one from the right, that were modulated at different frequencies. Using distributed source modeling of magnetoencephalography results, we estimate how spatially directed attention modulates the ASSR in neural regions across the whole brain. Attention enhances the ASSR power at the frequency of the attended stream in contralateral auditory cortex. The attended-stream modulation frequency also drives phase-locked responses in the left (but not right) precentral sulcus (lPCS), a region implicated in control of eye gaze and visual spatial attention. Importantly, this region shows no phase locking to the distracting stream. Results suggest that the lPCS in engaged in an attention-specific manner. Modeling results that take account of the geometry and phases of the cortical sources phase locked to the two streams (including hemispheric asymmetry of lPCS activity) help to explain why past ASSR studies of auditory spatial attention yield seemingly contradictory results.

## INTRODUCTION

The ability to focus attention on a sound of interest amidst irrelevant signals is vital for an animal’s survival. While the challenge of directing selective auditory attention, dubbed the “cocktail party problem,” is well recognized (Cherry, 1953), the neural mechanisms controlling it are poorly understood. In the current study, we took advantage of the ability of the cortex to phase lock to input acoustic oscillations around 40 Hz. By driving the auditory cortex with known frequencies, we explored what other cortical regions may be involved in attention. Here, we leveraged the excellent temporal resolution of magnetoencephalography (MEG) and the inherent responsiveness of the auditory cortices to sounds that are amplitude modulated at frequencies around 40 Hz (Mäkelä, 1987) to study the neural areas engaged in directing auditory spatial attention.

Auditory stimuli that are modulated at 40 Hz drive a strong, phase-locked auditory steady-state response (ASSR; Galambos et al., 1981). Numerous studies have established that the ASSR is robust in humans (Kuwada et al., 1986; Dolphin and Mountain, 1992; Aiken and Picton, 2008; Gockel et al., 2011). Moreover, the ASSR has been proposed for a range of applications, including characterizing sensorineural hearing impairments in clinical audiology and audiometry (Kuwada et al., 1986; Lins et al., 1996), quantifying maturation of top-down processes (Herdman, 2011), and monitoring the depth of general anesthesia (Plourde and Picton, 1990). The strength of the ASSR depends on the modulation frequency, and has strong modes around 40 and 80 Hz; it has also been shown to track multiple simultaneous stimuli modulated at different frequencies (Lins and Picton, 1995; Ross et al., 2000). The ASSR to a particular stimulus is sensitive to additions of new sounds to the acoustic scene even when there is no spectral overlap between the different sources (Ross et al., 2005b). Together, these two properties suggest that the ASSR can be used to “frequency tag” neural responses. Specifically, cortical responses locked to the modulation frequency of a particular stimulus in a scene must be related to processing of that stimulus.

In the psychoacoustics literature, attention is often thought of as operating as a gain control mechanism, enhancing the internal representation of the attended stream and suppressing the representation of the ignored streams (Lee et al., 2014). Consistent with this view, several studies using fMRI have demonstrated that BOLD signal activity in auditory cortical areas is modulated by attention (Grady et al., 1997; Petkov et al., 2004; Woods et al., 2009). In addition, attention robustly modulates event-related responses to sound (ERPs; Hillyard et al., 1998; Choi et al., 2013), and spectrotemporal features of speech are better represented in the cortical responses when the speech is attended to in a mixture compared to when it is ignored (Ding and Simon, 2012a). Similarly, the strength of the visual analog of the ASSR, the visual steady-steady response (VSSR) is modulated so robustly by top-down selective attentional processes that it has been proposed as a control signal for brain computer interfaces (BCIs, Kelly et al., 2005).

Analysis of how attention alters the ASSR may give insight into the mechanisms controlling selective listening, but only recently have studies attempted to take advantage of frequency tagging of acoustic stimuli to investigate auditory attention. Importantly, those studies that have explored how auditory attention modulates the ASSR have produced mixed results.

An early study reported that the ASSR was unaffected by attention (Dean Linden et al., 1987); however, others report some modulation of the ASSR by attention (Tiitinen et al., 1993; Müller et al., 2009). Indeed, attentional modulation of the ASSR has been suggested as a basis for BCI; binary classification based on ASSR yielded better than chance performance in assessing the direction that was attended (Kim et al., 2011). While some of these studies have found slightly more modulation on the left hemisphere than on the right hemisphere (Tiitinen et al., 1993; Müller et al., 2009; Kim et al., 2011), some have reported the opposite effect (Bidet-Caulet et al., 2007). Yet another study concluded that the way that attentional modulated the ASSR depended on the AM frequency and was asymmetric (Müller et al., 2009). One study investigating inter-modal attention (visual versus auditory) concluded that the attentional modulation of the ASSR exhibits a right hemispheric dominance (Saupe et al., 2009).

Importantly, the majority of the past studies of attentional modulation of the ASSR performed analyses on sensor/scalp EEG/MEG data and/or fit equivalent (cortical) current dipole (ECD) source models. Analysis in sensor/scalp space is difficult to interpret, as the observed signals are mixtures of sources that are correlated, driven by the common acoustic input. Depending on the phase relationships of the contributing sources, the observed mixture may show anything from an increase to a decrease in ASSR strength. Using ECD models also poses potential problems. Specifically, the ECD models all assume a pre-specified, small number of dipoles. If the dipole locations are assumed a priori, such analysis will not reveal sources unless they are near the assumed locations. When the dipole locations are free parameters, ECD analysis tends to only find the dominant sources that produce characteristic dipole-like fields. Moreover, the problem of determining dipole locations is a non-convex optimization problem; therefore, the solution tends to depend strongly on the “guesses” used to initialize search algorithms. Given these caveats, it is conceivable that inconsistencies in the conclusions of past studies exploring how attention modulates ASSRs arise because the analysis fails to identify all of the constituent sources that contribute to the observed results. Another point worth considering is that all of these studies used monaural or dichotic stimuli, thereby confounding asymmetry in the neural response with asymmetries in stimulus presentation.

To our knowledge, the current study is the first to use MEG to investigate auditory selective attention by analyzing ASSR data in cortical space using a whole-brain distributed source model (Dale et al., 2000), thereby eliminating assumptions about how many neural sources are present, or how their activity is distributed in space and over time. The current study also uses true binaural stimuli generated using head-related transfer functions (HRTFs; see Shinn-Cunningham et al., 2005), which reduces statistical differences in the acoustic presentation levels stimulating the left and right ears. One attention study that we have come across that analyzed ASSR responses in cortical space using a distributed inverse solution used EEG data to study the effects of inter-modal (visual versus auditory) attention (Saupe et al., 2009). The authors of the study suggested that attentional modulation of the ASSR may be asymmetric in the primary auditory areas. In addition, while the majority of studies localize generators of ASSR to or close to the primary auditory cortex or anatomically to the Heschl’s gyrus (Ross et al., 2005a; Bidet-Caulet et al., 2007), Saupe et al. (2009) also hinted that weak responses, and attentional modulations of these responses, can be found even in frontal areas. Owing to its insensitivity to the conductivity disparities between the layers of brain tissue, the skull, and the scalp, MEG has slightly better spatial resolution than EEG (Hämäläinen et al., 1993). Furthermore, the physics of the interaction between the fields produced by primary dendritic currents and the volume conducting brain tissue suggests that the sources to which MEG is most sensitive are complimentary to the sources to which EEG is most sensitive (Hämäläinen et al., 1993). Thus, our whole-brain approach using MEG and true binaural stimuli gives us an opportunity to identify any asymmetrically located sources, and to discover weaker sources beyond the dominant responses from primary auditory cortices that have not previously been identified. Hence, these methods may help to elucidate and reconcile the seemingly contradictory effects of attention on ASSR strength reported thus far.

The psychoacoustic literature argues that selective attention is directed toward perceptual “objects” (Bregman, 1990). In an acoustic scene, different objects are segregated from the mixture by grouping of perceptual features (e.g., pitch, location, timber, etc.), which are derived from acoustic attributes such as harmonic structure, binaural cues, and frequency content (Shinn-Cunningham, 2008; Shinn-Cunningham and Best, 2008). Electrophysiological correlates of object-based perception and attention have been recently found with MEG; the cortical representation of an attended stream was invariant to low-level manipulations such as masker level (Ding and Simon, 2012b). Perceptual objects usually correspond to tangible sources in the real world. Most naturally occurring sources excite multiple sensory modalities; for instance, a source typically conveys its physical location through both visual and auditory information. Given that attention is object based, it seems plausible to hypothesize that selective attention would engage cortical regions specialized for analyzing real-world attributes, such as location, in a modality non-specific manner. Here, we hypothesize that there exists a multimodal spatial attention network that operates on both visual and acoustic inputs. In vision, where sensory acuity decreases with the eccentricity from the fovea, circuitry controlling eye gaze is intimately tied to circuitry controlling spatial attention (Corbetta et al., 2008). Specifically, the frontal eye-fields (FEFs), part of the premotor cortex, both control eye gaze and participate in directing spatial attention even in the absence of eye movement (Bruce et al., 1985; Wardak et al., 2006). Imaging studies using fMRI show that auditory spatial attention tasks also activate the FEFs (Salmi et al., 2007; Wu et al., 2007; Lee et al., 2013). Broadman Area 8, which includes the FEF in primate, has anatomical projections to both auditory and visual association areas (Barbas and Mesulam, 1981). Thus, we hypothesized that FEFs would be engaged during spatially selective auditory attention.

To avoid some of the assumptions made in previous studies and to enable us to identify all neural regions involved in controlling auditory spatial attention, here we undertake a whole-brain analysis, employing appropriate conservative corrections for multiple comparisons. Based on previous work, we wondered if left and right FEFs would be driven with an ASSR during our auditory spatial attention task. To test for this specific possibility, we use an independent go/no-go saccade paradigm (described in Section “Auxiliary FEF Localizer Task”) to localize the FEFs, thus allowing us to determine whether this portion of the visuo-spatial attention network also is engaged during spatial auditory attention.

## MATERIALS AND METHODS

### SUBJECTS

Ten subjects (two female), aged 20–40, were recruited from the Boston University community. All had pure tone hearing thresholds better than 15 dB HL in both ears at octave frequencies between 250 Hz and 8 kHz. Subjects provided informed consent in accordance with protocols established at the Massachusetts General Hospital and Boston University. All subjects completed a training block of 20 trials. The training block was repeated until subjects responded correctly on at least 15 out of 20 trials. All subjects were able to meet this criterion within two runs.

### STIMULI AND TASK

**FIGURE 1:**
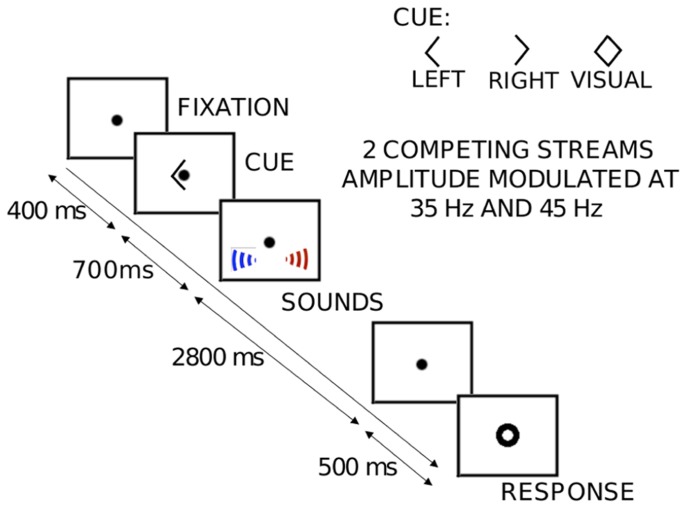
**An illustration of the sequence of events and stimuli presented in each trial of the auditory spatial attention task.** Each trial begins with the subjects fixating at the center of the screen on a dot. A visual cue (left arrow, right arrow, or diamond) indicates the attentional target (left “auditory” stream, right “auditory” stream or the center “visual” stream) to the subject. About 700 ms after the offset of the cue, the 2800-ms-long auditory streams begin, composed of spoken letters. In the “visual” trials (diamond cue), the center fixation dot flickers a fixed number of times as the letter streams are presented. The subjects count either the number of “E”s spoken from the target location (in the “auditory” trials) or the number of flickers (in the “visual” trials). 500 ms after the offset of the targets, a visual response circle is displayed to signal that it is time for the subjects to indicate their response with a button press (0, 1, 2, or 3). Crucially, the two competing auditory streams are amplitude modulated at different rates (35 or 45 Hz, assigned randomly).

**Figure 1** shows the layout of the sequence of events in each trial. Each trial consisted of two simultaneous but spatially separated sequences of seven spoken vowel tokens (recorded in house; the set consisted of the American pronunciations of the vowels A, E, I, O, and U). The speech tokens were monotonized to have a fundamental frequency of 183 Hz using PRAAT (Boersma and Weenink, 2012). Each recorded vowel was 400 ms long; these vowels were concatenated to create random sequences, each of duration 2.8 s. The sequences were digitized at 48.8 kHz and were spatialized using non-individualized HRTFs recorded binaurally using KEMAR (Shinn-Cunningham et al., 2005). Two different HRTFs were used, one for a source to the left and one for a source to the right, corresponding to locations at ±30° azimuth, elevation at eye level, and distance of 1 m. Competing left and right sequences were each amplitude modulated, but with different modulation frequencies (35 and 45 Hz). This allowed us to isolate and estimate ASSRs corresponding to each of the competing streams. All auditory stimuli were presented using the Tucker-Davis Technologies System 3 programmable audio processing hardware controlled using MATLAB (MathWorks Inc., Natick, MA, USA). All visual stimuli were controlled using the PsychToolbox MATLAB library (Brainard, 1997).

Each experimental session consisted of a total of 708 trials divided into eight blocks. Of these, 480 were “auditory” trials, while the remaining 228 were “visual” trials. In 320 of the 480 “auditory” trials, the two modulation frequencies were assigned randomly, one to the left stream and the other to the right stream. The visual trials presented auditory streams that were statistically identical to those presented in these auditory trials. In the other 160 “auditory” trials, the modulation frequencies were either switched midway from 35 to 45 Hz or vice-versa to allow us to assess behaviorally if the modulations are perceptible (and thus potential cues for directing selective attention).

Subjects were instructed to fixate on a center dot, which was on screen throughout the experimental session. At the beginning of each trial, there was a visual cue instructing subjects as to what task to perform on that trial; this cue began 700 ms before the onset of the streams. On both kinds of auditory trials, subjects were instructed to count the number of times the letter “E” appeared in the appropriate stream, a task that ensured that they maintained their attentional focus on the target stream throughout the sequence. On these trials, the visual cues signified that listeners should attend to either the left stream (left pointing arrowhead) or the right stream (right pointing arrowhead) and ignore the competing stream. On visual trials, the visual cue was a diamond (both left and right arrow heads presented simultaneously; see **Figure 1**), signaling that the subjects should ignore the sounds and instead report the count of the number of times the visual fixation dot flickered. None of the “auditory” attention trials contained a visual flicker.

Five-hundred milliseconds after the offset of the streams, subjects were presented with a “visual response circle,” signaling the time window within which they could indicate their response. The 500 ms delay between the end of the sound and the response period helped to temporally isolate neural activity corresponding to auditory processing from that corresponding to planning or executing the button press. On any given auditory trial, the correct count of letter Es was equally likely to be 0, 1, 2, or 3. On the visual trials, the correct flicker count was zero on 70% of the “visual” trials and 10% each for counts of 1, 2, or 3. The order of trials within the session was counterbalanced. Crucially, by design, the zero-flicker visual condition and the auditory conditions have identical stimuli; the only difference was in what the listeners were instructed to attend. The 160 (70% of 228) zero-flicker visual trials served as our visual control; the other visual trials were not analyzed further. Finally, the number of trials of each target type was equal, by design.

### DATA ACQUISITION, CONDITIONING, AND SOURCE LOCALIZATION

Magnetoencephalography data were acquired inside a magnetically shielded room (IMEDCO) using a MEG 306-channel dc-SQUID Neuromag Vector View system (Elekta-Neuromag) with 204 planar gradiometers and 102 axial magnetometers. Two bipolar electro-oculogram (EOG) electrode pairs measured horizontal eye movements and blinks. A bipolar chest electrode pair was used to record electrocardiogram (ECG) data. All data were recorded at a sampling rate of 600 Hz with a bandpass of 0.1–200 Hz. Four head position indicator coils were used to monitor head position (see, Liu et al., 2010; Lee et al., 2012). Samples containing artifacts associated with eye-movements and blinks were extracted by detecting peaks from the vertical EOG channel; samples with cardiac artifacts were similarly identified from ECG data. These samples were used to define spatial filters to help suppress artifacts using the signal-space projection method (Uusitalo and Ilmoniemi, 1997): one for blink artifact removal and another for cardiac artifact removal. Data were then low-pass filtered to 100 Hz. Finally, epochs were rejected if the peak-to-peak range over the epoch exceeded either 1000 fT in any magnetometer channels or 3000 fT/cm in any planar gradiometer channels.

For source localization and spatial normalization, two T1-weighted high-resolution structural magnetic resonance images (MRIs) were acquired during a separate session using a 3.0 T Siemens (Erlangen, Germany) Trio whole body high-speed imaging device equipped for echo planar imaging (EPI). We used a 3D multi-echo magnetization-prepared rapid gradient echo (ME-MPRAGE) sequence repetition time or (TR, 2530 ms; echo spacing, 7.25 ms) echo time, or (TE, 3 ms; flip angle 7°; voxel size, 1.3 mm × 1.3 mm × 1 mm). A 3D structural image was created for each participant by averaging the two MPRAGE scans after correcting for motion. The geometry of each participant’s cortical surface was reconstructed from the 3D structural MRI data using FreeSurfer software (http://surfer.nmr.mgh.harvard.edu). The segmented cortical surface was registered to an average cortical representation by optimally aligning individual sulcal-gyral patterns (Fischl et al., 1999). We employed a surface-based registration technique based on folding patterns because it provides more accurate inter-subject alignment of cortical regions than volume-based approaches (Fischl et al., 1999; Van Essen and Dierker, 2007). The cortical surface was decimated to a grid of 4098 dipoles per hemisphere, corresponding to a spacing of approximately 5 mm between adjacent source locations on the cortical surface. The MEG forward solution was computed using a single-compartment boundary-element model (BEM; Hämäläinen and Sarvas, 1989). The head-position information from the first run was used to estimate the sensor location relative to the source space. Sensor data from subsequent runs were transformed to correspond to the head-position of the first run using the signal-space separation method (Taulu et al., 2005). The cortical current distribution was estimated using minimum-norm estimate (MNE) software (http://www.martinos.org/mne); in this solution, we assumed that the orientation of the source was fixed and perpendicular to the cortical mesh. Cross-channel correlations in the recording noise used to calculate the inverse operator were estimated from data collected without a subject present (empty-room data). To reduce the bias of the MNEs toward superficial source distributions, we used a noise-normalization procedure to obtain dynamic statistical parametric maps (dSPMs) as *z*-scores (Dale et al., 2000).

### AUXILIARY FEF LOCALIZER TASK

A memory-guided go/no-go saccade task in the MEG was used to obtain a functional localization of individual frontal eye-fields (FEFs; for details about the saccade paradigm, see Lee et al., 2013). We focused on the FEFs located in and around the precentral sulcus and gyrus (Simó et al., 2005). For each subject, the anatomical constraints to the bilateral superior and inferior precentral sulci and the precentral gyri were defined by an automated surface-based parcellation (Fischl et al., 2004). Within these regions in the averaged group data, we functionally constrained the FEF-ROI to vertices showing activity (i.e., differences in dipole strengths) in the “go” versus “no-go” saccade contrast with a threshold of *p* < 0.05 following a conservative Greenhouse–Geisser non-sphericity correction. This contrast between the “go” and “no-go” trials isolates saccade-generating activity associated with the FEFs. This provided subject-specific spatial localization data for the FEFs to compare to our findings from the whole-brain analysis of the auditory spatial attention data.

### SPECTRAL ANALYSIS

**FIGURE 2:**
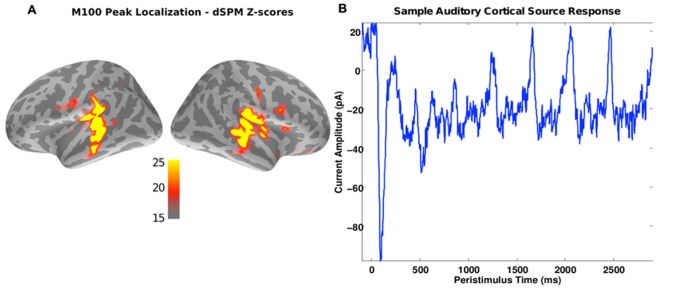
**(A)** A source image estimate of the M100 peak in response to the sound onset for a representative subject. Colors indicate the *z*-scores obtained using the dSPM method. As expected, the primary auditory cortices show up as “hot” spots in the image. The regions exceeding a *z*-score threshold of 20 served as ROIs for the spectral analysis of the ASSR response. **(B)** Source current amplitude time-course estimated using MNE [average of the strongest five sources in the “hot” spot in **(A)**]. Peaks in response to the individual digit onsets are evident every 400 ms.

For ROI analysis, the whole-brain dSPM scores were averaged over a window of 90–120 ms post sound onset, which aligns with the expected time of the stimulus-onset elicited M100 response (see **Figure 2B**). These average values were then thresholded at *z* > 20 to yield subject-specific primary auditory labels (see **Figure 2A**). Across subjects, the size of the largest contiguous cluster in the ROIs varied between 20 and 36 vertices in the left hemisphere and 20 and 42 vertices in the right hemisphere. Thus for further analysis, the strongest contiguous cluster of 20 sources (vertices) was used for each subject and hemisphere. Given that similar sensor noise levels (empty-room data use for noise-normalization) were observed across subjects, fixing the number of vertices in the source cluster ensured that the uncertainties (variances) of summary estimates (such as average spectral power in the ROI) were similar across subjects.

In order to evaluate if attention modulates the ASSR response in auditory areas, the data from the strongest 20 sources in each auditory ROI were entered into a spectral analysis. From each of the source vertices within the ROI, data were epoched from -50 to 2950 ms relative to the sound onset time. The spectral power at the stimulus “tag” frequencies of 35 and 45 Hz were estimated using the multi-taper method (Thomson, 1982), using three bi-orthogonal prolate-spheroidal sequences that minimized the spectral leakage outside of a bandwidth of 1.33 Hz (Slepian, 1978). The average power across the vertices in the auditory-onset-defined ROI was contrasted across different attention conditions.

In order to detect other cortical regions involved in auditory spatial attention (i.e., those not involved in the auditory onset response), we computed the phase-locking values (PLVs; the consistency of the phase of the response relative to the stimulus; e.g., see Lachaux et al., 1999) over the entire source space for each frequency bin. Because the PLV is a normalized metric (in each trial, only the phase of the response at the analysis frequency is used), it allows regions with low power but with responses phase-locked to a periodic stimulus to be detected. PLVs were computed using a bootstrap technique (Zhu et al., 2013); as a result, PLV estimates are approximately normally distributed, which then allows us to use *t*-tests (appropriately corrected for multiple comparisons) to be performed across the whole brain.

## RESULTS

### BEHAVIORAL RESULTS

Overall, performance on the auditory spatial attention task was significantly above the chance level of 25% (mean = 71%, SD = 8%; *p* < 0.0001). An analysis of incorrect responses revealed that the reported count was generally higher than the number of occurrences of the target letter in the attended stream (*t*-test, *p* < 0.02). Critically, performance did not depend on which stream the subjects attended (35, 45 Hz, or switched AM; repeated measures ANOVA with three levels, *p* = 0.6).

### AUDITORY ROI RESULTS

**Figure 2A** shows the M100 response, averaged over subjects. Activity is strong bilaterally in areas associated with auditory sensory processing, as expected. These areas were driven strongly over the duration of the auditory streams. This can be seen in the time course of the activity, shown in **Figure 2B** for an example subject. In addition to the very strong M100 response, there are positive deflections every 400 ms, following the onsets of the discrete vowel onsets making up each stream (at 0, 400, 800, 1200, 1600, 2000, and 2400 ms).

**FIGURE 3:**
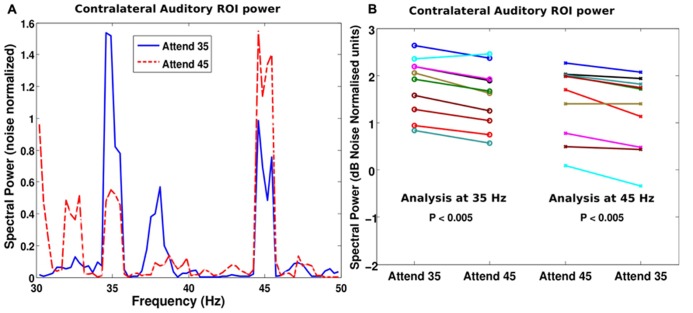
**(A)** Power spectrum of the contralateral auditory ROI for identical stimuli but different attentional targets for a representative subject. When the subject attended to the stream modulated at 35 Hz (blue solid line), the power at 35 Hz was higher than when the subject attended to the other stream modulated at 45 Hz (red dashed line). The analogous result was true at 45 Hz, i.e., when the subject attended the 45 Hz modulated stream, the power was higher than when the subject attended the 35 Hz stream (i.e., red dashed line is higher than the blue solid line at 45 Hz). **(B)** Similar effects were seen across our cohort of subjects. The spectral power at 35 Hz is plotted on the left half of the panel (open circles denoting individual subject values) and the spectral power at 45 Hz is plotted on the right half (crosses for individual subject values). When the subject attended to the stream modulated at 35 Hz, the 35 Hz power is higher for most subjects than when they attended to the 45 Hz stream. Similarly, the 45 Hz power was higher when the subjects attended to the 45 Hz stream rather than the 35 Hz stream. The effect, though small, was robust and consistent across subjects.

The auditory ROI defined using the M100 response was analyzed to evaluate if attention modulated the response. **Figure 3A** shows the power spectrum of the response in the auditory ROI from the hemisphere that was contralateral to the attended stream for a representative subject. When the subject attended to the stream tagged at 35 Hz, the response at 35 Hz was stronger than when he attended to the stream modulated by 45 Hz (blue solid line is higher than red dashed line at 35 Hz). The converse was also true: the 45-Hz response was stronger when the corresponding stream was attended than when it was ignored (red dashed line is higher than blue solid line at 45 Hz). This enhancement of the neural representation of the attended stream in contralateral auditory cortical areas was consistent across subjects (*p* < 0.001 at both 35 and 45 Hz; see **Figure 3B**). Indeed, all but one subject showed a stronger response at the tagged frequency (for both tag frequencies) when the corresponding stream was attended to than when it was not attended. This enhancement was also evident when the power at the tag frequencies was compared in the condition where that stream was attended and when both auditory streams were ignored and the subjects counted visual flashes (paired *t*-test: *p* < 0.05 at both 35 and 45 Hz). That is, the power at 35 or 45 Hz was higher when the subjects attended to the corresponding stream than when they ignored the auditory stimuli altogether in the control condition. There was no statistically significant difference (*p* = 0.2) in the power at the tag frequency of the unattended stream between the auditory attention and the count-flash conditions.

While both ipsi- and contralateral ASSR responses tended to be enhanced by attention, some subjects showed an asymmetry, i.e., more attention enhancement in the source contralateral to the attended hemifield; as a result of this, the ASSR from the source ipsilateral to the attended hemifield was not significantly enhanced by attention at the group level. Moreover, there were some asymmetries in the overall ASSR response itself (as opposed to modulations due to attention). Specifically, for many subjects, the right auditory ROI showed strong responses to both the left and the right streams (i.e., simultaneously to both 35 and 45 Hz), whereas the left ROI showed strong responses only to the contralateral source (i.e., the right stream). This is in line with the right auditory cortex dominance in spatial processing suggested by excision data, which show that right hemispheric lesions result in bilateral localization deficits whereas left hemispheric lesions sometimes produce no spatial deficits at all (Zatorre and Penhune, 2001). As a result of these complex interactions, overall, at the group level, the most robust effect of attention was an enhancement of the contralateral auditory source response (see **Figure 3B**).

### WHOLE BRAIN PLV RESULTS

**FIGURE 4:**
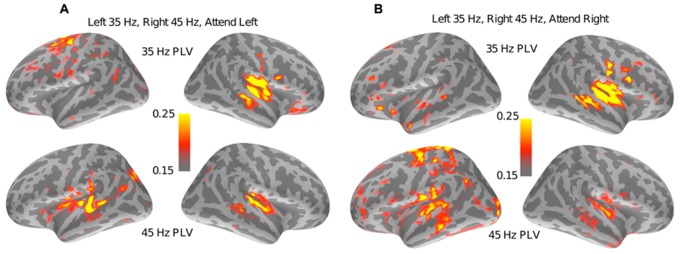
**Results of the whole brain PLV analysis for a representative subject.** **(A)** PLV values at both 35 Hz (top half) and 45 Hz (bottom half) are shown when the left stream is modulated at 35 Hz, the right stream is modulated at 45 Hz, and the subject is attending to the left stream. The superior left (but not right)-precentral sulcus shows PLV values well above the noise floor (of 0.11) at 35 Hz but not at 45 Hz. **(B)** PLV values for a stimulus that is identical to **(A)**, but with the subject now cued to attend to the stream modulated at 45 Hz. In this case, the superior left (but nor right)-precentral sulcus shows strong phase-locking at 45 Hz and not at 35 Hz. Taken together, the panels suggest that the left-precentral sulcus phase locks to the stimulus modulations in an attention-specific manner.

To detect other regions involved in auditory spatial attention, a whole-brain PLV analysis was performed (see Section “Spectral Analysis”). **Figure 4A** shows the results of the PLV analysis for a representative subject for the set of trials where the left stream (modulated at 35 Hz) was attended and the right stream (modulated at 45 Hz) was ignored. There were robust ASSRs at both 35 and 45 Hz for contralateral auditory sources. Interestingly, a region at the superior precentral sulcus also shows small but significant PLV at 35 Hz only, i.e., only at the frequency tag of the attended stream. **Figure 4B** shows the results of the PLV analysis for the same subject for identical stimuli but when the subject attended to the right stream, modulated at 45 Hz. As with **Figure 4A**, the auditory sources showed robust phase locking at the tag frequencies of the contralateral stream. However, the superior precentral sulcus now showed no phase-locking at 35 Hz; instead, there was significant phase-locking at 45 Hz when the 45-Hz-modulated stream from the right was being attended. The same effect was seen when the modulation frequencies were reversed and the left stream was modulated at 45 Hz and the right stream at 35 Hz. Notably, only the left precentral sulcus showed such attention-specific phase-locking.

**FIGURE 5:**
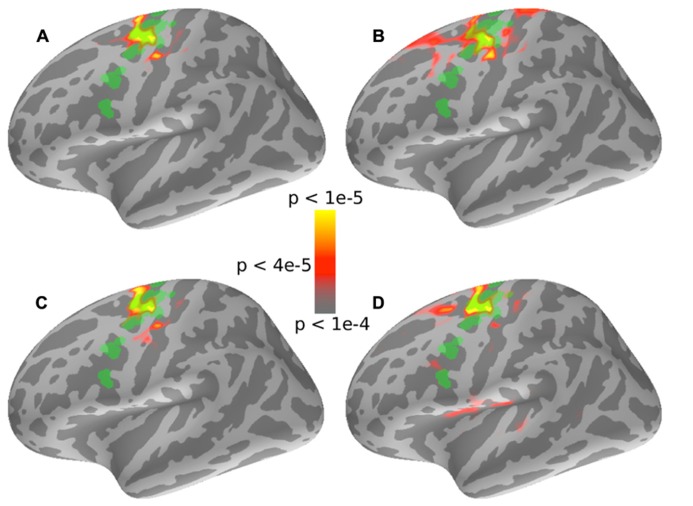
**Results of a whole brain comparison of PLV values between the “auditory” attention conditions and the “visual” control conditions for different stimuli and attentional cue combinations.** In all four cases, the superior left-precentralsulus sulcus shows PLV values significantly greater in the “auditory” attention condition than for the “visual” control conditions. Note that there were no significant regions in the right hemisphere in any of the four cases (not displayed here). The localization of the left-FEF obtained from the auxiliary saccade task is shown overlaid in green for visual comparison. **(A)** and **(B)** show 35 Hz PLV contrasts when the subjects attended to the 35 Hz stream, which was either presented in the left or the right hemifield, respectively. **(C)** and **(D)** show 45 Hz PLV contrasts when the subjects attended to the 45 Hz stream for the stream presented in the left or right hemifield, respectively.

In order to test if this effect was robust, the whole-brain PLV values for the attention condition were contrasted with the whole-brain PLV values for the count-flashes condition using a paired-*t*-test. The *t*-maps were then thresholded to limit the false-discovery rate to *q* = 0.05 to allow for multiple testing (Benjamini and Hochberg, 1995; Benjamini and Yekutieli, 2001). In all four cases (2 tag frequencies × 2 locations), the left-precentral sulcus showed a significantly higher PLV at the tag frequency in the attention condition compared to the count-flashes control condition with identical stimuli (see color-map results in **Figure 5**).

We wished to compare the location of the significant attention-specific phase-locking in the precentral sulcus found by our whole-brain PLV analysis to the localization of the FEF obtained from the auxiliary saccade task. We therefore overlaid the FEF ROI defined by the saccade task onto the PLV contrast map (see **Figure 5**, FEF ROI shown in green). The precentral sulcus that was phase-locked to attended, but not unattended sound streams (for the same physical input stimuli) overlapped significantly with the FEF ROI defined by the saccade task. Moreover, the region that was significantly phase-locked to the attended source was very similar across the four cases (2 tag frequencies × 2 locations; compare color maps in the four panels of **Figure 5**). Interestingly, the auditory attention condition only evoked phase-locked activity from the left precentral sulcus; there was no significantly phase-locking in the corresponding region in the right hemisphere (results not shown).

## DISCUSSION

This study is the first, to our knowledge, that uses a whole-brain distributed source localization procedure to assess the effects of auditory attention on the ASSR, that also uses true binaural stimuli using HRTFs as stimuli when studying attention effects on the ASSR. Our results show that the ASSR from the cortical source contralateral to the attended hemifield is enhanced in a frequency-specific manner. Moreover, using a whole brain PLV analysis with our frequency tagging design, we implicate the left FEF in controlling auditory spatial attention.

### DISTRIBUTED SOURCE VERSUS ECD/SENSOR-SPACE ANALYSES

Previous studies using VSSR (the visual analog of the ASSR) report robust changes in phase-locked responses due to attention; that is, the modulation in a given source drives the neural signal more strongly when that source is attended compared to when the same source is ignored. In contrast, ASSR studies report mixed results. In the current study, we find that though the modulation of the ASSR by attention is small, it is robust and detectable. This may be a direct consequence of our choice of stimuli, which allows for asymmetrical overall ASSR activity to be discovered, and the fact that we adopted a whole-brain analysis rather than relying on assumptions about what regions are likely to be engaged during auditory attention tasks, which allows for weak sources beyond the primary auditory cortices to be discovered.

Because of the spatial spread of electric and magnetic fields generated by neural sources, sensor-space measurements are a mixture of multi-source activity. If attention modulates the response from one such neural source differently than it affects others, the observed effect on the ASSR in the scalp sensors would depend on how the different sources sum at the scalp. How the different sources sum in turn depends on the geometry of the sources, the configuration of sensors, and the electrical properties of the tissue between source and sensor (Hubbard et al., 1971; Okada et al., 1997). This is the kind of result we found, with the auditory source responding more strongly when the contralateral source is attended, but left FEF (lFEF) responding only to the attended source, regardless of where it is located. Thus, because attention alters the responses to the auditory and lFEF sources differently, the net effect on the response observed on the scalp may vary widely across listeners, even though the effect of attention on the individual sources is qualitatively similar across subjects. Specifically, there are considerable individual differences in head and brain geometry, which could lead to inter-subject inconsistencies in what is observed if analysis is done in sensor space, obscuring consistent source-level effects at the group level.

To try to ameliorate the effects of field spread, some studies have used ECD analysis, assuming a pre-specified small number of sources (Williamson and Kaufman, 1981). While this procedure can help to un-mix sources, the estimated source currents are known to be particularly sensitive to misspecification of the number of dipoles (Hämäläinen and Ilmoniemi, 1994). Specifically, if experimenters fail to model some of the sources that are phase-locked to the input, it can bias the estimates of the sources that they do include in their model.

In our study, we found that the left FEF produces phase-locked responses to the attended auditory stimulus, but not to the unattended auditory stimulus. We also found that auditory sensory areas respond strongly, and that this activity is stronger when the contralateral stream is attended compared to when it is ignored. These two effects can interact in complex ways. Though the amplitude of the phase-locked response at the FEF is very small compared to the strength of the response from the auditory sources, the responses may be large enough to obscure the small attentional modulations in the ASSR coming from auditory cortex.

We used a PLV analysis that fixes the response magnitude at unity for each trial to reveal regions producing small but phase-locked responses to the stimulus. We find that the spectral power of the FEF source is small compared to the spectral power of the auditory source (about 15 dB less on average), so that the net effect of this source on the total response observed at the sensors will tend to be modest. Of course, it is possible to evaluate this more quantitatively. To determine how this consistent but relatively weak FEF source could influence results, we explored what our results would look like for an ECD-based analysis that included only two dipoles corresponding to the dominant, auditory source (one per hemisphere). For a given subject, the estimated attentional modulation of the auditory source found in such an analysis depends on the phase relationship between the lFEF source and the auditory source and the overlap of their lead-fields in the measurements. Because the active lFEF dipole is not included in the simple two-dipole model, the estimated activity in auditory cortex will be influenced by the activity in lFEF. Analytically, this bias in the current amplitudes of the auditory sources is given by:

$$\overset{\wedge }{q}=q-c\,q^{\prime }$$

$$c=g^{T}\,h/g^{T}\,g$$

where $\overset{\wedge }{q}$ is the estimated strength of the auditory dipole *q*, *q*′ is the dipole corresponding to the lFEF source, *g* and *h* are the lead-fields of dipoles *q* and *q*′, respectively, and *c* gives the correlation between *g* and *h*.

**FIGURE 6:**
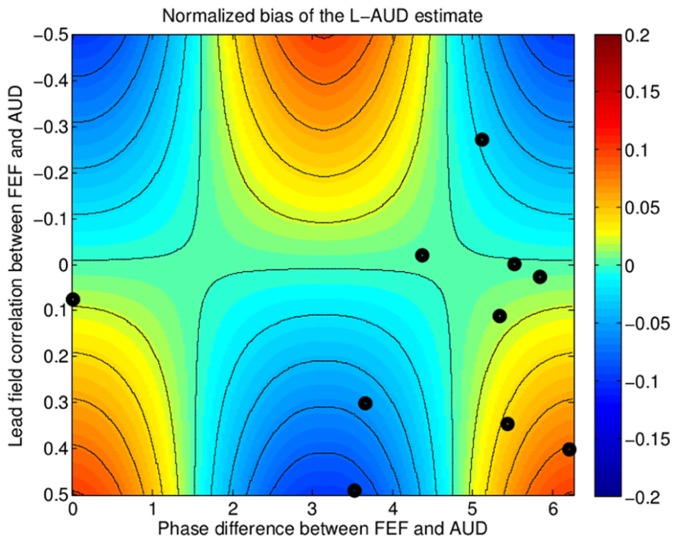
**Simulation of the bias in source strength estimation due to model mis-specification, i.e., fitting only two auditory (AUD) sources instead of three (two AUD and one FEF).** The bias depends on two parameters: (1) the overlap (inner product) between the lead-fields of the missing source and the fitted sources, plotted along the horizontal axis, which varies across subjects due to differences in brain geometry, and (2) the phase relationship between the FEF response and the AUD source response, plotted along the vertical axis. The normalized bias (relative to the true auditory source strength) in the estimated activity from auditory cortex is shown by the color-map, with hot colors indicating a positive bias (overestimation of auditory cortex activity) and cold colors indicating a negative bias (underestimation of auditory cortex activity). Estimates of the parameter values from the individual subject data are shown as black dots overlaid on the colormap. Depending on the geometry and the phase-relationship, the strength of the auditory source estimate can be biased either positively or negatively.

In order to illustrate the effect of this model misspecification on the estimate of the auditory source, we simulated the bias in the ASSR current estimate as a function of (1) the phase difference in the response between the frontal source and the auditory source, and (2) the lead-field correlation *c*; both of these parameters varied across subjects, based on our whole-brain analysis. The level of the lFEF activity was fixed at 15 dB below the auditory source amplitude, similar to the relative amplitudes of the estimated sources we found in our whole-brain analysis. The results of the simulation are shown as a function of the two free parameters in the simulation in **Figure 6**, where the color map denotes the bias (over-estimating auditory source strength in warm colors, under-estimating the strength in cool colors). To illustrate the kinds of variability that might be expected in real observations, we show the individual subject estimates of the phase difference between the FEF and auditory sources and the correlation as points overlaid on the plot. We see that depending on the brain geometry of the individual subject, the “extra” FEF source that is not accounted for in the model could either increase, have no effect on, or decrease the estimated current amplitude at the auditory source. Moreover, the increases and decreases in the estimates of the auditory source due to the not-modeled FEF source are of the same order of magnitude as the actual change in the auditory source strength due to attention. In other words, by erroneously assuming a too-simple, two-dipole model, an experiment may not only overlook the FEF source engaged by attention, but also fail to see the attentional modulation of the auditory source that is being modeled. To understand this effect better, we performed a simple statistical analysis to see whether we would be able to observe a consistent across-subject effect of attention on the auditory source estimated from the two-dipole model. Because of the bias in the auditory source estimate caused by the FEF activity, the effect of attention on the ASSR response of the modeled auditory source fell below the threshold for statistical significance (*p* = 0.14, paired *t*-test). Thus, for the data we obtained in the current experiment, attentional modulation of the auditory source estimated from the two-dipole auditory-source model would be missed because the FEF source bias causes inconsistent results across subjects.

### BINAURAL VERSUS DICHOTIC STIMULI

We used true binaural stimulation, simulating our spatial auditory streams using HRTFs. As a result, both the attended and the unattended speech streams were delivered to both ears. For the source locations we simulated, the interaural level differences in the signals were relatively small (on the order of 5 dB); interaural time differences and spectral cues were the primary cues for localization. Because of this, the overall level of the stimuli presented to the two ears was essentially equal; any asymmetries in neural responses must arise out of physiological asymmetries, given that there is no asymmetry in the stimulation of the ears. Although previous studies have reported asymmetries in the attentional modulation of the ASSR (Bidet-Caulet et al., 2007; Müller et al., 2009), some of these results are confounded by the fact that the studies used dichotic stimulation, which results in asymmetries in the acoustic presentation. While we find a general right hemispheric dominance of the ASSR itself, at the group level, we did not find any asymmetries in the effect of attention; the auditory cortical ROI contralateral to the attended hemifield consistently showed enhancement of the ASSR response whether the attended location was to the left or the right (also see Lazzouni et al., 2010).

### FREQUENCY TAGGING AS A TOOL FOR PROBING ATTENTION

The current study successfully used frequency tagging to examine the effects of attention on the neural representation of different auditory streams in the scene. The technique proved powerful, especially combined with a whole-brain analysis. However, it is worth discussing whether the amplitude modulation imposed on the speech streams had any perceptual consequences that could have confounded our conclusions. Anecdotally, subjects reported that although there was an increased “roughness” to the stimuli compared to speech tokens without amplitude modulation, they could not tell that the two streams had different modulation frequencies. Moreover, the subjects reported that they could clearly understand the spoken letters despite their perceptual roughness. As reported above, the majority of response errors overestimated the count of Es, a result that suggests that errors were most often due to hearing extra target letters, which were present in the to-be-ignored stream, rather than failures of intelligibility. Together, these results suggest that the imposed modulations did not interfere with understanding the acoustic inputs.

Crucially, we found that there were no differences in performance depending on what the tag frequency of the attended stream was. Indeed, performance was the same even when the modulation frequency switched mid-way through the streams. Given that performance was unaffected by the modulation (including a switch in modulation midstream), it seems safe to say that the location of the vowels in the stream was the dominant cue used to focus attention on the stream of interest, and that the imposed modulation did not disrupt spatial attention.

In sum, although the modulation altered the quality of the speech streams, it did not affect their intelligibility. Not only did subjects subjectively report that they could not tell the two modulation frequencies apart, discontinuities in modulation frequency had no effect on performance. Thus, we believe that this kind of frequency tagging might be a useful tool, both in exploring how listeners control attention, and even as a way to drive brain-computer interfaces, similar to how VSSRs are being used (Middendorf et al., 2000). Indeed, for some locked-in patients who have trouble controlling eye gaze, ASSR-based BCI may prove more practical than a VSSR-based device.

### FEF INVOLVEMENT IN AUDITORY SPATIAL ATTENTION

Our results demonstrate that the FEFs, which are involved in eye gaze control and visuospatial attention, are also engaged when listeners direct spatial auditory attention. Activity in left FEF is robustly (albeit relatively weakly) phase-locked to the attended stimulus, but not to the unattended stimulus. Moreover, this asymmetric left (but not right) FEF activity is present regardless of whether attention is directed toward a source in the left or the right hemifield. While it is difficult to directly compare our results to results from studies using a other imaging modalities, previous fMRI studies demonstrate that activity in the left dominated fronto-parietal network is enhanced during attentionally demanding trials compared to fixation, both during visual and during auditory tasks (Giesbrecht et al., 2003; Slagter et al., 2007; Hill and Miller, 2010). One recent MEG study also found left dominance in top-down auditory spatial attention, with left FEF showing enhanced activity during spatial attention both in preparation for upcoming stimuli and during their presentation (Lee et al., 2013).

At first glance, the left lateralization we observed here seems at odds with classic reports of “hemispheric dominance” in visual studies, where the right hemisphere processes information from both visual fields, whereas the left exclusively encodes the right visual field (Mesulam, 1981). One factor that may help account for this difference is the contrast we used in our study. Specifically, we contrasted conditions in which the acoustic stimuli were identical; only the attentional task differed. Because of this feature of our experimental design, our results emphasize regions engaged in purely top-down control. Consistent with this view, left FEF may be part of a dorsal network controlling volitional attention, while right FEF may be more engaged during exogenous attention and attention shifting (Corbetta et al., 2008).

Previous studies that have shown FEF involvement in spatial attention tasks have had to contend with the possible confound that small eye movements (micro-saccades), which are a natural response to directing spatial attention, may result in FEF activity; the FEF activity may not reflect effects of auditory attention *per se*. In order to ameliorate the likelihood of this explanation for FEF activity, past studies often used high-resolution eye tracking to rule out eye movement explanations. Here, we take a completely different approach. Because we analyze frequency-specific steady-state responses, it is extremely unlikely that eye movements caused the FEF activity that we see; it is hard to argue that any gaze shifts would be phase-locked to the 35–45 Hz modulation in our acoustic stimuli. Our approach also has the advantage that the activity associated with motor preparation (generating a response button press) is unlikely to be frequency-specific to 35 or 45 Hz in an attention-specific manner.

The frequency-specific phase-locked activity seen at the FEF could be a consequence of the distributed-inverse solution procedure employed (typically referred to as the point spread), arising from the under-constrained nature of the MEG/EEG inverse problem. However, the inverse operator used is linear. For the specific contrast employed (attention minus control), the attention effects we found in the auditory cortex (i.e., responses to a contralateral attended source are enhanced) cannot explain why phase-locking to the attended source was present in left FEF, regardless of which hemi-field the attended source was in, without any such effect in right FEF. Moreover, despite the small increase in the ASSR power in the auditory cortex, the PLV contrast between attention and control conditions does not show any differential auditory cortical activity. Thus, the observed lFEF phase locking cannot be a consequence of point spread.

It is surprising that acoustic-stimulus phase-locked activity travels as far upstream as the frontal executive control areas. This activity may be a consequence of functionally specific communication between the lFEF and the auditory cortical regions. Indeed, Area 8, the anatomical region that includes the FEF in primates, is known to have direct projections to auditory association areas (Barbas and Mesulam, 1981). Perhaps the preparatory activity associated with lFEF (Lee et al., 2013) in anticipation of the auditory stimulus establishes a functional link between the stimulus-driven activity in the auditory cortex and the FEF, similar to top-down control of visual spatial attention (Awh et al., 2006). Regardless of whether the phase-locked response in the FEF is directly responsible for the attentional enhancement of the auditory cortical ASSR or an indirect consequence of functional connectivity between sensory auditory areas and executive control regions, our results implicate left FEF in directing auditory spatial attention. This is consistent with the view that attention is object based and that real objects are inherently multimodal. Further, our results support the view that there is a multimodal spatial attentional-control network that is closely linked with directing eye gaze.

## Conflict of Interest Statement

The authors declare that the research was conducted in the absence of any commercial or financial relationships that could be construed as a potential conflict of interest.
